# Semi-automated digital measurement as the method of choice for beta cell mass analysis

**DOI:** 10.1371/journal.pone.0191249

**Published:** 2018-02-06

**Authors:** Violette Coppens, Gunter Leuckx, Yves Heremans, Willem Staels, Yannick Verdonck, Luc Baeyens, Nico De Leu, Harry Heimberg

**Affiliations:** 1 Beta cell Neogenesis, Vrije Universiteit Brussel, Brussels, Belgium; 2 Collaborative Antwerp Psychiatric Research Institute, Faculty of Medicine and Health Sciences, University of Antwerp, Campus Drie Eiken, Antwerp, Belgium; 3 University Department of Psychiatry, Campus Duffel, Duffel, Belgium; 4 Department of Pediatrics, Division of Pediatric Endocrinology, Ghent University, Hospital and Department of Pediatrics and Genetics, Ghent, Belgium; 5 Department of Endocrinology, Universitair Ziekenhuis Brussel, Brussels, Belgium; 6 Department of Endocrinology, Algemeen Stedelijk Ziekenhuis Aalst, Aalst, Belgium; Centre de Recherche des Cordeliers, FRANCE

## Abstract

Pancreas injury by partial duct ligation (PDL) activates beta cell differentiation and proliferation in adult mouse pancreas but remains controversial regarding the anticipated increase in beta cell volume. Several reports unable to show beta cell volume augmentation in PDL pancreas used automated digital image analysis software. We hypothesized that fully automatic beta cell morphometry without manual micrograph artifact remediation introduces bias and therefore might be responsible for reported discrepancies and controversy. However, our present results prove that standard digital image processing with automatic thresholding is sufficiently robust albeit less sensitive and less adequate to demonstrate a significant increase in beta cell volume in PDL versus Sham-operated pancreas. We therefore conclude that other confounding factors such as quality of surgery, selection of samples based on relative abundance of the transcription factor Neurogenin 3 (Ngn3) and tissue processing give rise to inter-laboratory inconsistencies in beta cell volume quantification in PDL pancreas.

## Introduction

Curative strategies for diabetes aim to restore a functional beta cell mass. A controlled increase in beta cell numbers provides an attractive alternative to the current clinical practice of beta cell transplantation. We reported beta cell mass expansion in adult mice by activation of multipotent progenitor cells and proliferation of pre-existing and newly formed beta cells upon tissue injury by pancreatic partial duct ligation (PDL) [1, 2]. However, strong controversy remains with regard to the beta cell volume in PDL pancreas: while some reported a 2- to 3-fold increase in beta cell volume [1–3] others found no such increase [4, 5].

After PDL, the size of the ligated pancreas decreases substantially compared to Sham-operated pancreas due to massive acinar cell loss [1]. Calculating a relative area fraction within tissues with non-comparable size will give biased results. More specifically, the beta cell area will be overestimated in PDL versus Sham-operated pancreas. Therefore, only absolute tissue area and, by extrapolation, tissue volume quantification will give reliable results concerning beta cell abundance. Based on Cavalieri’s principle [6], the absolute beta cell volume can be quantified by multiplying the sum of the beta cell plane areas, measured on (immuno)stained tissue sections and systematically sampled throughout the entire pancreas, with the distance between 2 sequential analyzed sections. Absolute tissue area (and cell numbers) can also be calculated by overlaying (immuno)stained tissue section images with a point grid (point counting method). The number of points falling within the area of the tissue of interest is then quantified and multiplied by the area plane represented by one such point [7].

Unfortunately, this procedure is very labor intensive and time consuming. In addition, manual interpretation of (immuno)staining to identify the area of interest is prone to inter-observer variability [8]. Recent advances in technology allow full-automatic area measurement and particle quantification at much higher throughput and avoid subjective bias or inter-observer variations. While fully automated, computerized morphometrical analysis is widely used in diverse research fields, both concordances [9] and discrepancies [10] with manual data acquisition have been reported. In reports that failed to detect an increase in beta cell volume upon PDL [4, 5], morphometry was performed by automatic software-based analysis without manual micrograph artifact correction or without submission of post-analysis output files for peer revision. In contrast, we observed increased beta cell volumes following manual point counting [1] and therefore hypothesized that fully automatic, non-verified analysis may result in biased findings, thereby contributing to the reported inconsistencies regarding the anticipated increase in beta cell volume and number in PDL pancreas. We employed standard NIH-developed ImageJ software to compare fully automatic versus manually-verified and corrected analysis of beta cell volume.

## Materials and methods

### Mouse manipulation

All animal experiments were performed according to the guidelines of our institutional "Ethical Committee for Animal Experiments" (ECD 12-595-5) and national guidelines and regulations. Pancreatic partial duct ligation or Sham operation was performed as described [1, 11] on 8 weeks old, male Balb/c mice.

### Immunostaining and image acquisition

Pancreas tails were harvested at day 14 after PDL or Sham operation, fixed overnight at 4°C in 10% neutral-buffered formalin, processed overnight in a Leica Histokinette processor and embedded in paraffin. The entire PDL or Sham pancreas was cut into serial 5μm thick sections, spaced apart by 150μm. For analysis, every 30th section was stained. This represents 3% of the entire PDL or Sham tail portion of the pancreas, sufficient to perform analyses with a relative error of <10% [11]. After dewaxing and rehydration, sections were incubated overnight at 4°C with a guinea pig anti-insulin antibody (1/1000, Diabetes Research Center, Brussels, Belgium). Bound antibody was detected with a cyanine-2-labeled secondary antibody (Jackson Immuno-Research, Suffolk, UK). Nuclei were stained with Hoechst 33342 (Sigma-Aldrich, St. Louis, MO, USA).

Whole-section images were captured at 20-fold magnification (NA: 0.45) as a stack with a monochromatic image for each channel using a Nikon Eclipse TE2000-E microscope equipped with a Marzhauser Tango stage for large image microscopy with a fixed exposure time of 15msec for insulin and 10msec for Hoechst. Individual 20X image fields where automatically stitched with 5% overlay, resulting in a whole-section 8-bit image.

### Beta cell morphometry

Image processing was performed with Fiji, an open-source distribution of ImageJ (version 1.48d), developed at the National Institutes of Health, USA and allowing user extensibility via Java plugins [12].

Detection of the whole-tissue area: In a first step, an ImageJ macro was created to automatically select the Hoechst^+^ area (S1c Fig) on each section as a representation of total tissue region of interest (ROI). This ROI selection is based on intensity-thresholding that was performed with the default Fiji automatically determined threshold (S1c Fig). To result in a confluent tissue ROI, the *Edit—Selection—Enlarge* function was applied to first enlarge and subsequently again shrink the automatic tissue ROI each by 30 pixels, resulting in inclusion of the area between adjacent nuclei (tissue boundary ROI, S1d and S1e Fig). In manually verified analyses of tissue labeled with Hoechst and immunostained for insulin, the tissue boundary ROIs were subsequently corrected on color images merged from the Hoechst and insulin channel for false-positive signals (S1e and S1f Fig, **asterisks**) as well as for adipose or fibrous tissue and lymph nodes, which were manually deselected based on morphologic identification by respective lower and circle-shaped higher density of nuclei (corrected tissue ROI) (S1e and S1f Fig, **arrowheads**). All ROIs are available for peer evaluation.

Detection of insulin^+^ area: The automatically determined or manually verified tissue boundary ROIs were applied to the insulin image for automatic or investigator-validated insulin area quantification respectively. All signals outside of the tissue boundary ROI (which is by definition not specific as it falls outside of tissue boundaries) was deleted by use of the *Edit—Selection—Clear Outside* function before proceeding to insulin ROI selection (S1h Fig, **arrows**). Within the boundary ROI, Fiji automatic or manually-set fixed thresholds were applied to select immune-stained insulin signal (auto- and fixed- threshold insulin ROI respectively) (S1i Fig). The fixed threshold was chosen as the median threshold that correlated most accurately with the insulin-specific signal in 3 random fields of 3 random sections in 4 samples (2 Sham, 2 PDL). To result in a confluent insulin boundary ROI, the *Edit—Selection—Enlarge* function was applied to first enlarge and subsequently again shrink the automatic tissue ROI each by 10 pixels (insulin boundary ROI, S1j and S1k Fig). In manually verified analyses, the insulin ROIs were corrected for false-positive and false-negative signal which were manually deselected or included respectively. Manual verification was performed by checking the conformity of each separate ROI with the corresponding insulin^+^ area on the composite red-green-blue (RGB) image. First, the monochromatic single insulin and Hoechst images were automatically composited in an RGB picture. The automatically generated auto-threshold insulin ROIs were then added as an overlay to the RGB picture. If a discrepancy was found between the ROI and the insulin^+^ area, the ROI was manually corrected by elimination of false-positive signal or by adding sub-threshold insulin^+^ areas (corrected insulin ROI, S1l Fig, **hatched arrows**). All ROIs are available for peer evaluation.

Beta cell and tissue volume quantification: for whole tissue plane area calculation, the plane area of the manually verified and automatically determined boundary ROI were measured.

For insulin volume calculation, the insulin image was made binary and the plane area of insulin boundary ROIs of particles exceeding a diameter of 6.5×10^−5^ mm (empirically defined lower limit for mean beta cell diameter; in correspondence with the cut-off used by Chintinne et al. [5]) was measured. Per sample, the total sum of the respective whole tissue and insulin areas were multiplied with the distance between 2 sequential analyzed sections (150μm) for volume extrapolation [13]. Of each analysis, an output file was generated and saved for peer evaluation. This output file can easily be overlaid on the RGB image to confirm islet outlines and trace the individual particles that are numbered during analysis (S1m and S1n Fig).

Data were analyzed by one-way and two-way ANOVA’s, power diagnostics and Levene’s test for equality of variances using JMP^®^ Pro 13.0.0 software (SAS Institute Inc.; Cary, NC, USA) and presented as mean±standard deviation.

## Results

### Fully automatic beta cell volume quantification is sufficiently robust to overcome data skewing due to micrograph artifacts

A 2- to 3-fold increase in beta cell volume in PDL versus Sham-operated pancreas was reported by several groups [1–3] but not reproduced by others [4, 5]. Xu et al. [1] analyzed the beta cell volume manually by the point counting method, while those who did not detect an increase in beta cell volume or number after PDL [4, 5] used automatic software-based analysis. To clarify the basis of the discrepancy, we performed automatic beta cell area quantification using digital image analysis software based on the same principles as described in other papers [4, 11]. Highly regarded within the worldwide scientific imaging community as the most elaborate and user-friendly scientific image processing program [12, 14], we used Fiji, a streamlined version of the open source ImageJ software, developed by the National Institutes of Health for all micrograph analyses.

As the contribution of Ngn3^+^ progenitors to beta cell neogenesis increases in parallel with the relative abundance of Ngn3 transcripts following PDL [2], we analyzed samples with Ngn3 transcript-levels between 36 and 68% of the level in duodenum, ranging from moderate to strong induction of Ngn3 gene expression after duct ligation. Three percent of the total PDL or Sham pancreas tail, sufficient for analysis with a relative error of <10% [5, 11], was immunostained for insulin to visualize beta cells. Hoechst 33342 was used to label cell nuclei. Pictures of entire tissue sections were taken with the 20-fold objective on a microscope able of taking large scale-type images (S1a Fig). The tissue boundary was determined by creating a ROI after automatic thresholding on the Hoechst image (S1b Fig) and making the ROI confluent using script-driven automatic ROI enlargement and shrinkage by 30 pixels (tissue boundary ROI; S1c–S1e Fig). Within tissue boundaries, ROIs of insulin^+^ areas were created for each section using automatic thresholding on the anti-insulin Cy2 channel, after which ROIs were made confluent (auto-threshold insulin ROI, S1i–S1k Fig).

**Fig 1:**
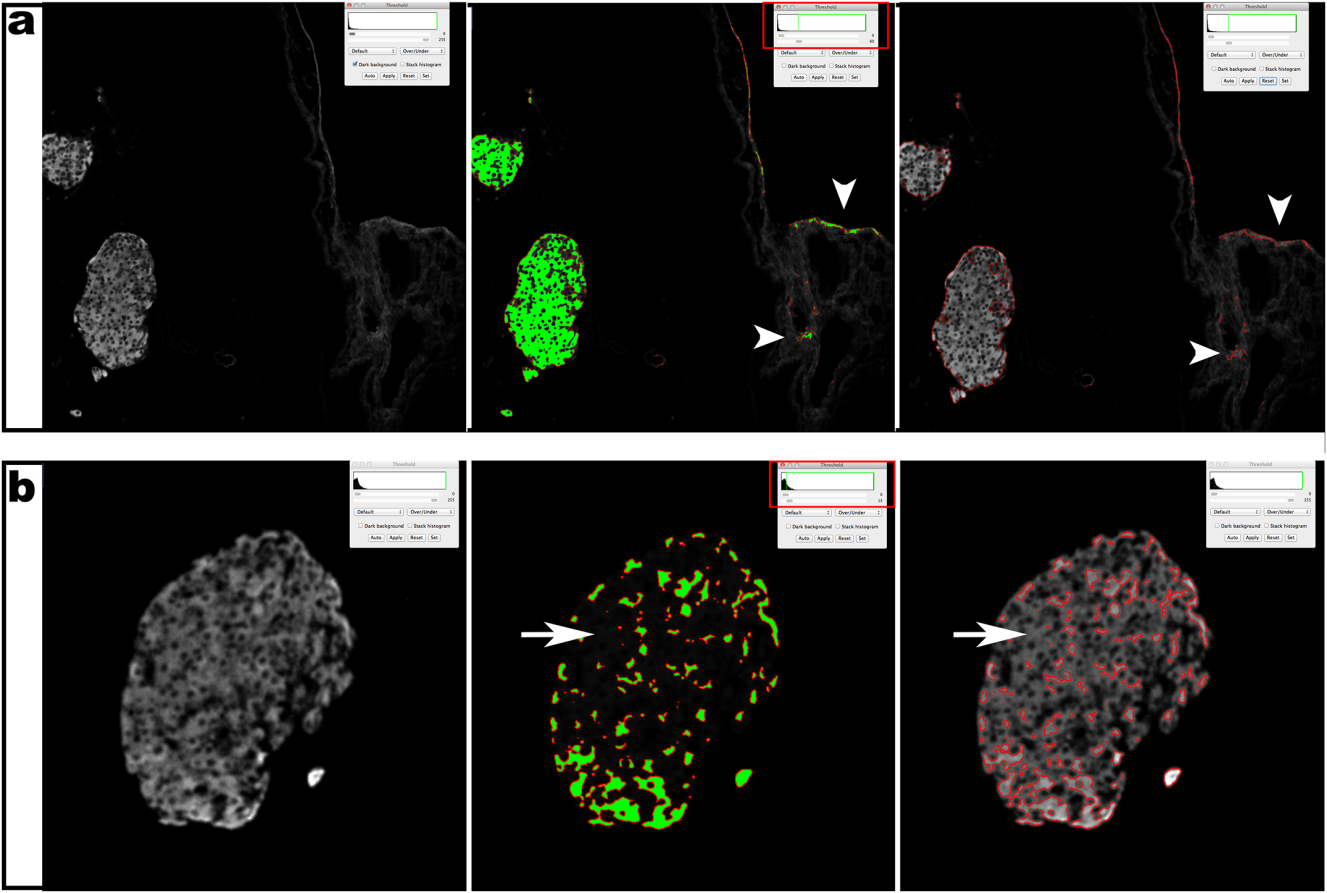
Fully automated analysis without manual data verification of insulin^+^ area results in both inclusion of noise and exclusion of weak positivity. (a) Inclusion of false positive signal (“noise”, white arrowheads). (b) Exclusion of weak positivity (white arrows). Left: raw image; middle: ROI (red) with automated threshold (green); right: ROI (red) on raw image.

**Fig 2:**
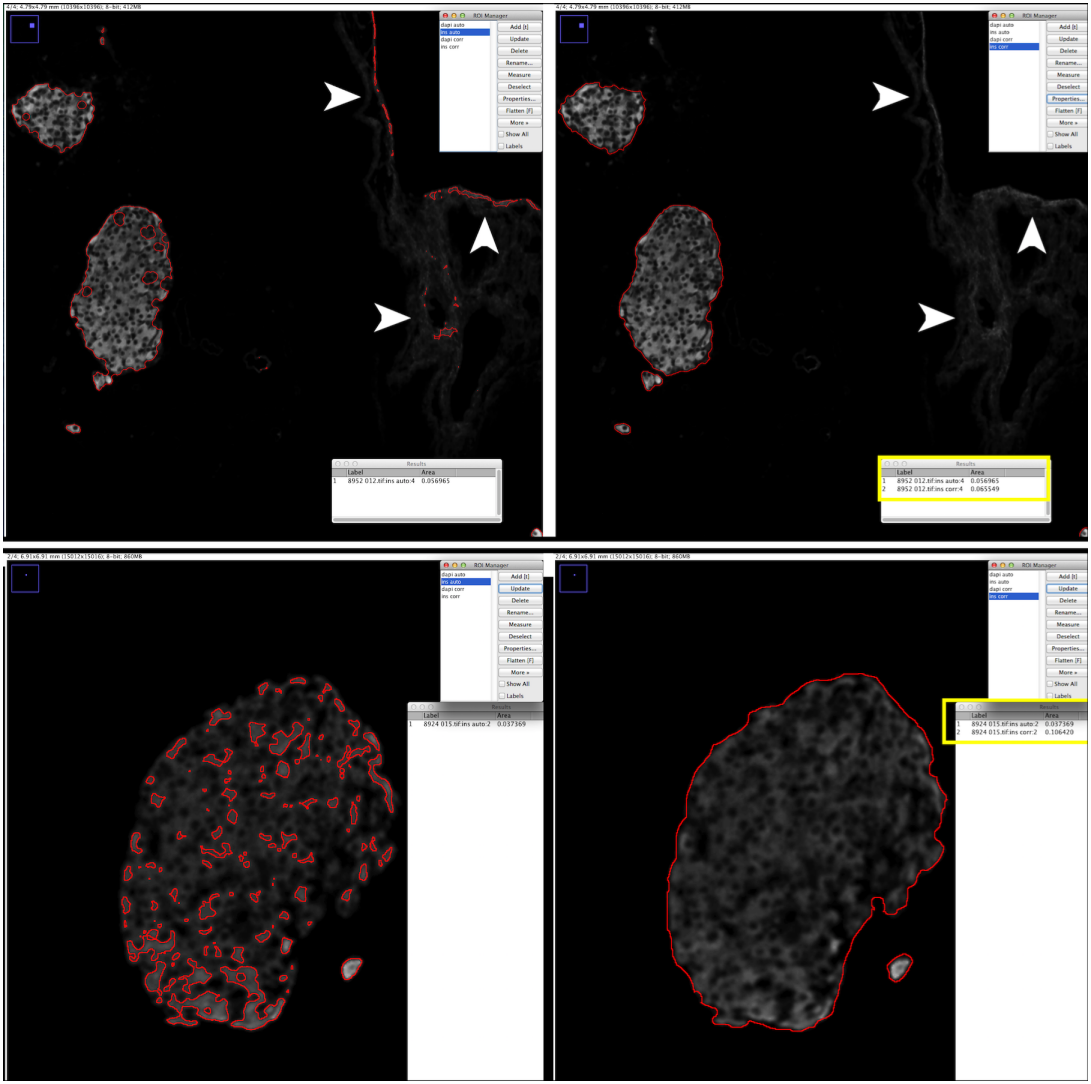
Depiction of manual correction of false-positive and false-negative signal. (a) Manual correction of false-positive noise (white arrowheads). (b) Manual correction of false-negative weaker signal (compare red encircled areas on both panels). Left: automatic threshold ROI, right: manually corrected ROI. ROI Manager: the blue highlight indicates the depicted ROI: highest ROI value: automatic insulin area (ins auto), lowest ROI value: corrected insulin area (ins corr). The yellow boxes demonstrate the difference in plane area before (ins auto, 1) and after (ins, corr, 2) manual ROI correction: **a** ins auto: 0.056965 mm^2^, ins corr: 0.065549 mm^2^; **b** ins auto: 0.037369 mm^2^, ins corr: 0.106420 mm^2^.

Visual inspection of these automatically generated insulin ROIs revealed that they often did not correspond to genuine insulin^+^ cells. We noticed both inclusion of false-positive signal (Fig 1a) as well as exclusion of weak insulin^+^ cells (Fig 1b). As these micrograph artifacts likely influence the beta cell volume quantification, insulin ROIs were manually corrected on all images by inclusion of false-negative signal to and exclusion of false-positive signal (corrected insulin ROIs) (Fig 2). Subsequently, the total plane area encompassed by either the auto-threshold or corrected insulin ROIs was measured per image and per sample and multiplied with the section thickness (5μm) and with the number of skipped sections (30) in between consecutive analyzed sections to extrapolate to total tissue beta cell volume (BCV) [13]. To investigate whether the analysis method (automatic vs. corrected) influenced BCV quantification in PDL vs. Sham pancreas, a 2x2 factorial analysis of variance (ANOVA) was performed with "analysis method" and "condition" (PDL vs. Sham) as independent variables. While the overall model showed a significant effect on variance (F = 12.45; p<0.0001), surprisingly, only the variable "condition" proved to be significantly contributing to inter-group variance (t = -6.07: p<0.0001), with no significant effect of either "analysis method" or an interaction effect of "analysis method" x "condition". This refutes our initial hypothesis that automatic quantification might be the causative factor for reported discrepancies in BCV increase in PDL-injured pancreas.

**Fig 3:**
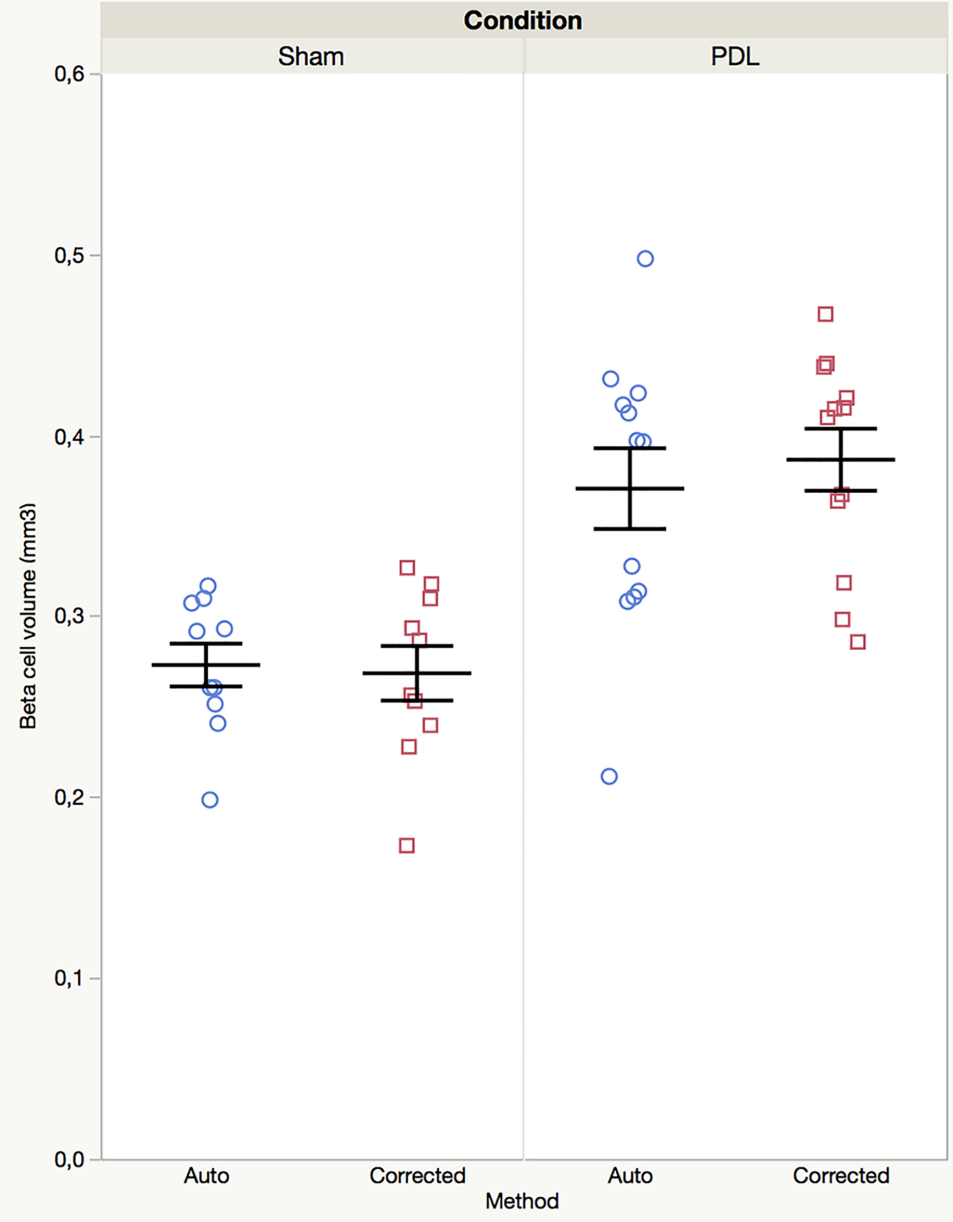
Beta cell volume quantification on non-corrected (Auto, circles) and corrected (Corrected, squares) insulin+ ROI’s. Investigator remediation of micrograph artifacts is not required to demonstrate a significant increase in BCV in PDL (right, n = 12) vs. Sham (left, n = 10) pancreas (2X2 ANOVA shows significant effect of condition but not of method nor of the interaction condition x method—F ratio of model = 12.45, p < 0.0001; t ratio of "Condition" = -6.07, p < 0.0001).

We hereby confirm our lab’s previous results by demonstrating a 1.5 increase in BCV in PDL versus Sham pancreas in both the investigator verified (0.39±0.06mm^3^, n = 12 vs. 0.27±0.05mm^3^, n = 10) and automatically analyzed samples (0.37±0.08mm^3^, n = 12 vs. 0.27±0.04mm^3^, n = 10) (Fig 3). In addition, this increase is sufficiently robust to overcome immunostaining micrograph artifacts.

### Beta cell volume quantification after investigator remediation of micrograph artifacts is the preferred method with regard to both statistical solidity and ethical considerations in animal experimentation

To investigate the individual statistical characteristics of the automated vs. corrected quantifications, separate one-way ANOVA’s were performed for both analysis methods. Levene’s test for equality of variance revealed that for the automated analysis method, variances were unequal between Sham (standard deviation (SD) = 0.037) and PDL (SD = 0.077) (F ratio = 5.73, p = 0.0266), while micrograph correction remedied inter-group inequality of variance (SD Sham = 0.047, SD PDL = 0.059; F ratio = 0.98; p = 0.3341). Reducing within-sample variation in the corrected analysis resulted in a more reliable statistical difference between Sham and PDL BCV (F ratio = 25.70, p <0.0001) compared to non-corrected, automatic analyses (Welch ANOVA allowing for unequal standard deviations, F ratio = 14.92, p = 0.0013). We hypothesized that such a considerable reduction in within-group inter-sample variation might have an influence on experiment power. Therefore, power analyses were performed for both the automated and the corrected analysis, using the standard deviations of the PDL groups as these were larger than those of both Sham groups (0.077 vs. 0.037 for the automated and 0.059 vs. 0.047 for the corrected analysis) with an alpha level of 0.05 and a predetermined power of 0.80. Interestingly, performing investigator remediation of micrograph artifacts allows for an impressive one-third reduction in sample size with a calculated total of 21 samples (i.e. 11 per condition) in the automated vs. 14 samples (i.e. only 7 per condition) in the corrected group.

## Discussion

We previously reported a doubling in beta cell volume as well as in insulin content in PDL versus Sham pancreas [1, 2]. However, the reproducibility of these findings has been debated [4, 5]. Immunostaining protocols are long since established and largely unified among the scientific community while the use of computerized image processing in data analysis is a fairly recent and discordant approach. We therefore questioned whether the latter could underlie the observed discrepancies. We observed that automatic thresholding of microscopy images can result in inclusion of false-positive signal. Increased occurrence of false positivity in the ligated pancreas is likely due to extensive injury-induced alteration of tissue morphology including massive acinar cell death (potentially causing non-specific binding of antibodies to dying and dead cells), influx of immune cells (potentially causing non-specific binding of antibodies to Fc-receptors on immune cells) and remodeling of the extracellular matrix (potentially causing electrostatic interaction of highly charged antibodies to connective tissue components with reciprocal charge) [15].

During our analysis, a greater variation in insulin signal intensity was observed in PDL pancreas as compared to Sham pancreas. Although automatic thresholding is accepted as the standard method in digital image processing, it is especially prone to underestimation of weaker positive signals by categorizing lower intensity pixels representing genuine insulin positivity as background. Fundamentally, automatic thresholding relies on the range of pixel intensities present in each individual image. Depending on the distribution of these pixel intensities and image depth, the threshold will shift. We conceive these variations in measured signal intensity to be at least partially caused by immunostaining and imaging. When staining entire sections, it cannot be guaranteed that every part of every section is covered with an equal concentration of antibody. This might result in slight differences in fluorescent signal intensity throughout the section. In addition, when imaging entire sections via stitch composition (especially with higher magnification objectives), the sections will be illuminated for extended periods of time, with the last section on the slide undergoing the longest light exposure. These areas will thus photobleach [16] more extensively than areas or sections imaged first, again resulting in heterogeneity of signal intensity throughout and between sections. A non-exclusive explanation for the variation in insulin signal intensity among individual beta cells is the described heterogeneity in insulin secretion within islets and throughout different pancreatic regions [17, 18]. Heterogeneity in the beta cell population concerning insulin production and secretion capacity [11] will inevitably result in varying amounts of primary antibody target throughout the tissue section, potentially making beta cells with lower insulin content at the moment of tissue processing fall beneath the automatic threshold-determined detection limit. Consequently, we reasoned that using full-automatically determined and, therefore, incorrect ROIs to quantify the total number of beta cells may result in erroneous data.

We manually verified and corrected the ROIs generated by automatic thresholding to ascertain that ROIs more truthfully represented the total beta cell area. Measuring the insulin^+^ areas represented by these corrected ROIs confirmed that beta cell volume does significantly increase in PDL versus Sham pancreas. To facilitate observer verification of automatically generated ROIs, we performed digital microscopy imaging using a 20x objective (NA: 0.45). We strongly believe that a magnification of at least 10-fold is required to adequately distinguish and include subthreshold single or small beta cell clusters. Future work will reveal whether the use of a 5-fold objective as used by Rankin et al. [4] further contributes to underestimation of beta cell area.

Statistical analysis of our data showed that fully automatic and manually corrected quantification both measured a 1.5-fold increase in BCV in PDL- versus Sham-operated pancreas, in agreement with our previously reported data [1, 2]. This finding excludes the method of analysis as main cause of inconsistency with regard to published BCV data in PDL pancreas. Differences may be due to other inter-laboratory variables such as quality of surgery, tissue processing and selection of samples with moderate to strong induction of Ngn3 gene expression following PDL. To provide complete transparency, we reported our methodology in a video article [11] and we invite interested scientists to our lab for hands-on PDL training. Although full-automatic analysis in a sufficiently large sample size (n = 10–12 per group) robustly demonstrates a significant increase in BCV in PDL vs. Sham pancreas, statistical analysis shows that the use of corrected micrographs results in a stronger statistical effect and in increased experimental power. In addition, corrected analysis allows for a reduction in the number of experimental animals by 50%. This finding supports the 3R (reduction, refinement, replacement) recommendations for ethical conduct of animal experimentation described in the Basel declaration [19]. Based on our data, beta cell morphometry after micrograph correction rather than fully automated quantification is the recommended method to measure BCV in PDL pancreas from both a scientific and an 3R point of view. We assume that our observations are not exclusively associated with the image processing program used (Fiji), but that they may also apply to other image processing programs using automatic thresholding for ROI determination (Volocity, IP Lab, …).

Scientists using computerized technologies for research analysis should check if these “silent” technology-inherent flaws are avoided. Therefore, we embedded automatic generation and saving of output files in the Fiji analysis script and provide access to all our raw data, analysis scripts, ROIs and output files for peer revision. We propose to always include the generation of these output files in automatic script-based digital analysis and to perform conscientious verification thereof to reveal errors and allow for rectification of biased data before publication. In addition, these files can be placed at disposition for peer review in case of debate regarding results or methodology.

In conclusion, we advocate caution towards scientific reports that do not mention manual verification and correction of automatically generated morphometry data. Delivery of insight in the methodology and verification used in tissue volume and cell number quantifications is strongly encouraged.

## Supporting information

S1 Fig Step-by-step illustration of the method used for beta cell volume quantification. (TIF)
