# Supplementary figures and images for: Semi-automated digital measurement as the method of choice for beta cell mass analysis
Source: PLoS One. 2018 Feb 6;13(2):e0191249. doi: 10.1371/journal.pone.0191249 (PMC5800540; doi:10.1371/journal.pone.0191249)

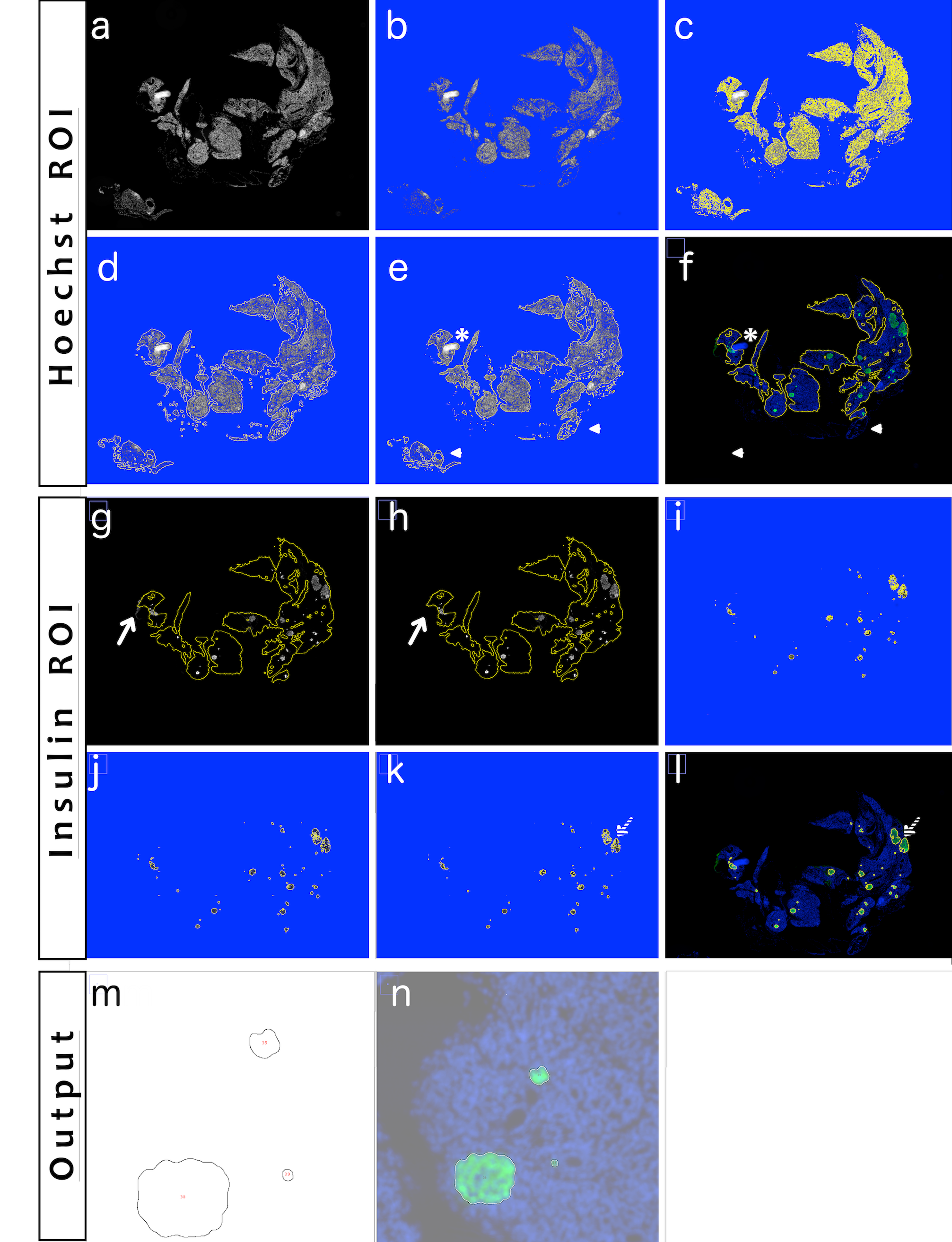

Supplement: S1 Fig — (TIF) [file pone.0191249.s001.tif]
